# Acute and long-term costs of 268 peripheral nerve injuries in the upper extremity

**DOI:** 10.1371/journal.pone.0229530

**Published:** 2020-04-06

**Authors:** Konstantin D. Bergmeister, Luisa Große-Hartlage, Simeon C. Daeschler, Patrick Rhodius, Arne Böcker, Marius Beyersdorff, Axel Olaf Kern, Ulrich Kneser, Leila Harhaus

**Affiliations:** 1 Department of Hand, Plastic and Reconstructive Surgery, Burn Center, BG Trauma Center Ludwigshafen, Plastic and Hand Surgery, University of Heidelberg, Heidelberg, Germany; 2 Department of Surgery, Clinical Laboratory for the Restoration of Extremity Function, Medical University of Vienna, Vienna, Austria; 3 Department of Plastic, Aesthetic and Reconstructive Surgery, University Hospital St. Poelten, Poelten, Austria; 4 Faculty of Social Work, Health, and Nursing, Hochschule Ravensburg-Weingarten, Weingarten, Germany; University Hospital Zurich, SWITZERLAND

## Abstract

**Background:**

Peripheral nerve injury in the upper extremity is linked to high socioeconomic burden, yet cost-analyses are rare and from small cohorts. The objective of this study was to determine the costs and long-term socioeconomic effects of peripheral nerve injuries in the upper extremity in Germany.

**Methods:**

We analyzed data of 250 patients with 268 work-related upper extremity nerve injuries from acute treatment to long-term follow-up on rehabilitation, sick-leave and disability-pension.

**Results:**

Patients were on average 39.9±14.2 years old, male (85%) and mean inpatient treatment was 7±6 days. Location of nerve was 8% (N = 19) proximal to the wrist, 26% (N = 65) at the wrist and metacarpus, and 66% (N = 166) at phalangeal level. Acute in-patient treatment for (single) median nerve injury accounted for 66% with hospital reimbursement of 3.570€, ulnar nerve injury for 24% and 2.650€ and radial nerve injury for 10% and 3.166€, all including finger nerve injuries. The remaining were combined nerve injuries, with significantly higher costs, especially if combined with tendon 5.086€ or vascular injury 4.886€. Based on location, nerve injuries proximal to the wrist averaged 5.360±6.429€, at the wrist and metacarpus 3.534±2.710€ and at the phalangeal level 3.418±3.330€. 16% required rehabilitation with average costs of 5.842€ and stay of 41±21 days. Sick leave was between 11–1109 days with an average of 147 days with socioeconomic costs of 197€/day, equaling on average 17.640€. 30% received a mean yearly disability pension of 3.187€, that would account to 102.167€ per lifetime.

**Conclusion:**

This large German patient sample indicates that nerve injury has a major impact on function and employment, resulting in significant health care costs. Both proximal and distal nerve injuries led to long-term disability, subsequent sick-leave and in 30% to permanent disability pension. These data are determined to support future studies and health economical work on prevention, treatment and rehabilitation of these often small injuries with great consequences.

## Introduction

Upper extremities are the most frequently injured body part and its peripheral nerves are easily severed by cutting or other traumatic injuries[1]. Consequently, the hand, our primary tool for environment interaction, is handicapped with potentially long-lasting deficits in a patient population that is generally young and healthy[2, 3].

Despite modern reconstructive surgery, in a significant number of patients a substantial loss of function remains[2–5]. This results from the key issues of slow or impaired nerve regeneration leading to delayed reinnervation with consecutive muscle fibrosis or painful neuromas at the site of injury. This is particularly prominent in proximal injuries, where the long regeneration distances between lesion and target organ represent a limiting factor for sufficient reinnervation[6–8]. Thereby results a considerable functional disability in the mostly young and previously healthy adult patients[2, 3, 9]. Consequently, nerve injuries are associated with long periods of recovery, sick leave and sometimes life-long functional disability[2, 10]. This is especially pronounced in work-accidents, where hands are easily injured during manual labor and thus life-long injury compensation from health-care providers may result.

So far, there are only cost analyses from small patient cohorts of Sweden and Switzerland, but none from a German collective [10, 11]. For this purpose, we analyzed data of treatment, rehabilitation and long-term compensation for work related accidents from the workers’ compensation termed the “German Social Accident Insurance” (Deutsche Gesetzliche Unfallversicherung) [12]. The aim of this system is to provide optimal treatment to those injured while working and ensuring their ability to return to work. In this study, we analyzed data on a unique set of 250 consecutive German patients treated for 268 nerve injuries. To our knowledge, it thus provides the first complete analyses of injury data, treatment costs, duration of rehabilitation and long-term socioeconomic results including life-long compensation for remaining health deficits in a large patient cohort. This analysis is therefore the biggest patient collective showing the substantial long-term effects and costs of nerve injuries of the upper extremity.

## Methods

Ethical approval was obtained from the German Federal ministry of work and social affairs (AZ: Iva1-41735-75) to obtain all patient data from the German Social Accident Insurances, without the need for additional approval of individuals. Therefore, and due to the anonymous patient data analyses, no individual patient consent was sought. Patients were identified by ICD Code (G56.2, G56.3, S54.0, S44.0, S64.0, G56.3, S54.2, S44.2, S64.2, S54.0, S54.1, S64.1, S44.1, S44.4) and/or OPS search (5–044.1–4, 5–045.1–4, 5–046.1–4, 5–047.1–4, 5–048.1–4, 5–049.1–4, 5–0580.1–4, 5–051.1–4, 5–052.1–4, 5–053.1–4, 5–054.1–4, 5–055.1–4). Thereby, all patients with work-related traumatic nerve lesions of the upper extremity between 2012 and 2016 treated at the BG Trauma Center Ludwigshafen were included in the analysis. Included were primary nerve injuries located from shoulder to the fingertip, without brachial plexus injuries. Secondary nerve injuries following fractures or iatrogenic nerve injuries were excluded. Cost refunding was according to standard health treatment compensation of the German Social Accident Insurance system, which is based on the diagnosis related groups system (DRG). Cost-analyses therefore present the sum of revenues that the system provided for the treatment of patients. Patient data acquisition was done by two independent reviewers (KB, LGH) from the hospital information system in a pseudonymized manner. Data on follow up was provided on request by the German Social Accident Insurance on all included patients. This was the reason for exclusion of private injuries, since the health insurances do not provide follow-up data for research causes. The follow-up for post-acute treatment was at least 78 weeks following injury, which is considered the standard for work-related accidents in Germany. At this time, patients undergo evaluation if they suffer from a substantially reduced ability to work as a consequence of the work accident. If the patient was still undergoing treatment at that time, the follow-up was subsequently longer. The CHEERS (Consolidated Health Economic Evaluation Reporting Standards) Checklist was used as a guide for data reporting and manuscript design [13].

### Statistics

All data analyses were conducted using Microsoft Excel in a two-stage manner by KB, LGH, SD and LH. Statistical analyses were conducted using SPSS Statistics Version 21 (IBM, USA). For descriptive statistics, the mean and the standard deviation were calculated for variables. Further statistical comparison between groups was conducted using either Student’s T-Test or Kruskal Wallis Test, see specifications in parentheses. A two-sided p value < 0.05 was considered to indicate statistical significance. The chosen alpha level for all tests was 0.05 and thus p < 0.05 was considered as statistically significant.

## Results

### Study characteristics

**Fig 1:**
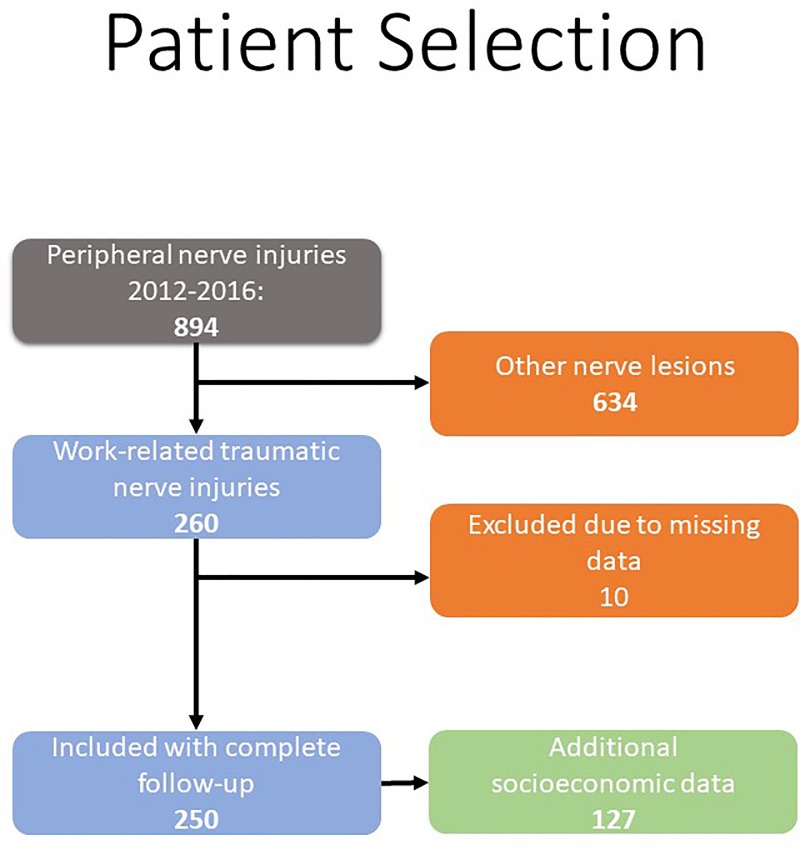
Flow-chart of patient selection: Selection and analysis of patients for this study.

Overall, 894 patients with both, non-work-related and work-related acute nerve injuries were identified from the database. Thereof, 634 were non-work-related injuries and ten had not completed their treatment or were lost to follow-up and thus excluded. Thereby, a total of 250 patients with 268 nerve injuries resulted. Data on post-acute treatment modalities was available from 127 patients from the responsible branch of the German Social Accident Insurance for all patients with a minimum of 78 weeks of follow-up, which is the standard time frame for work compensation (Fig 1).

### Demographics

A total of 250 patients with 268 nerve injuries resulting from work accidents were included, of which 212 were male (85%). At time of injury, patients were on average 39.9±14.2 years old, with males being on average 40.4±14,4 and females being 36.9±12.1. Employment relationship was 86% employed (N = 215), 5% self-employed (N = 13) and 2% students (N = 5). In 7% (N = 17) of the included patients, the employment relationship was not specified. The majority of patients were employed in the wood and metal industry (21%, N = 52), followed by construction (15%, N = 37) and trade industry (14%, N = 35), and thus the greater part working in manual labor professions.

### Injury

**Fig 2:**
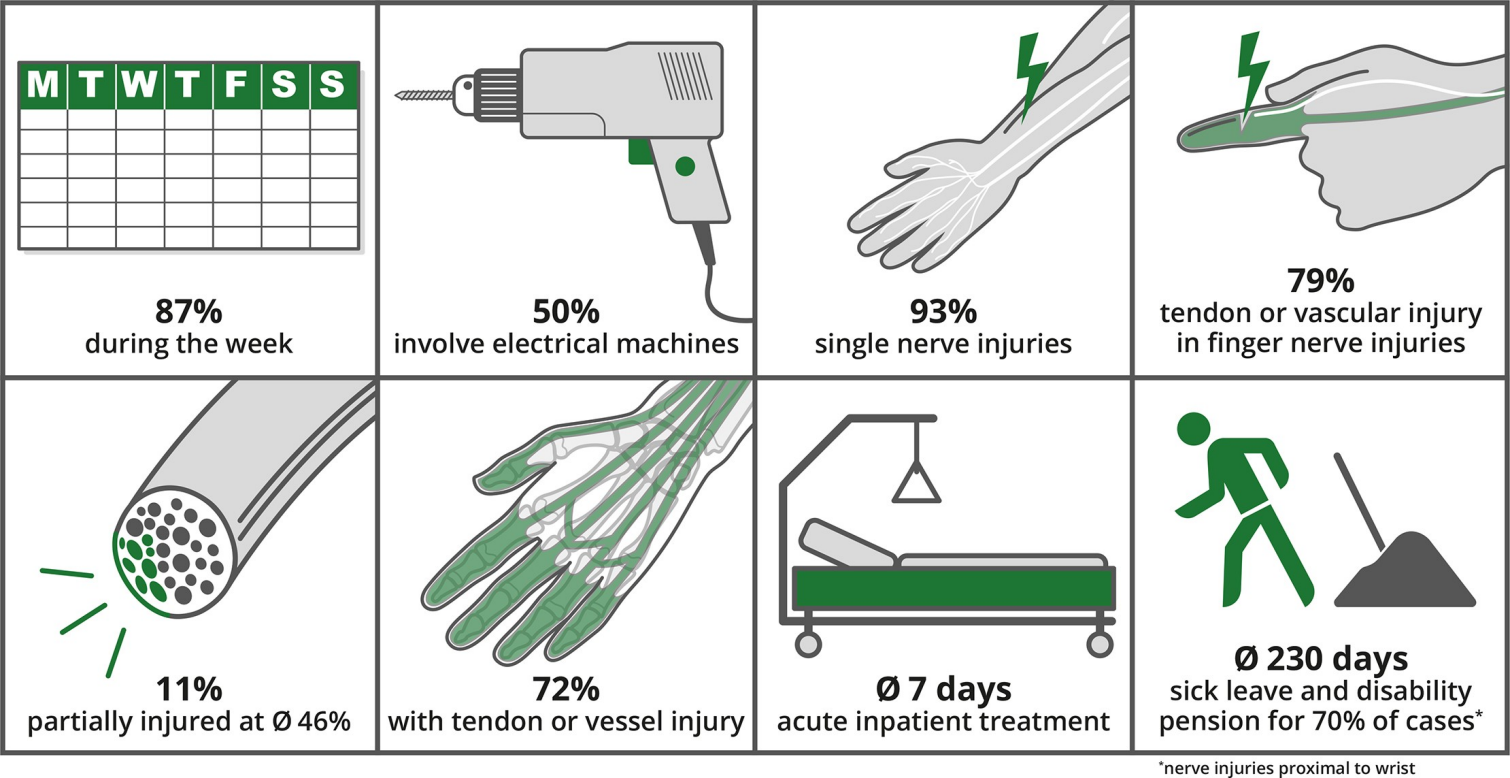
Injury characteristics: Schematic illustrations of the main injury characteristics of the involved patient population.

The majority of patients were injured (87%, N = 217) during the work week (Monday to Friday). Open injuries were dominant (94%, N = 234), while closed injuries represented 5% (N = 14). In 86% foreign objects were involved in the injury, most dominantly electrical machines 36%, glass 15% or knives 13%. Electrical machines were responsible for 50% of cases with more than one injured nerve (Fig 2).

**Fig 3:**
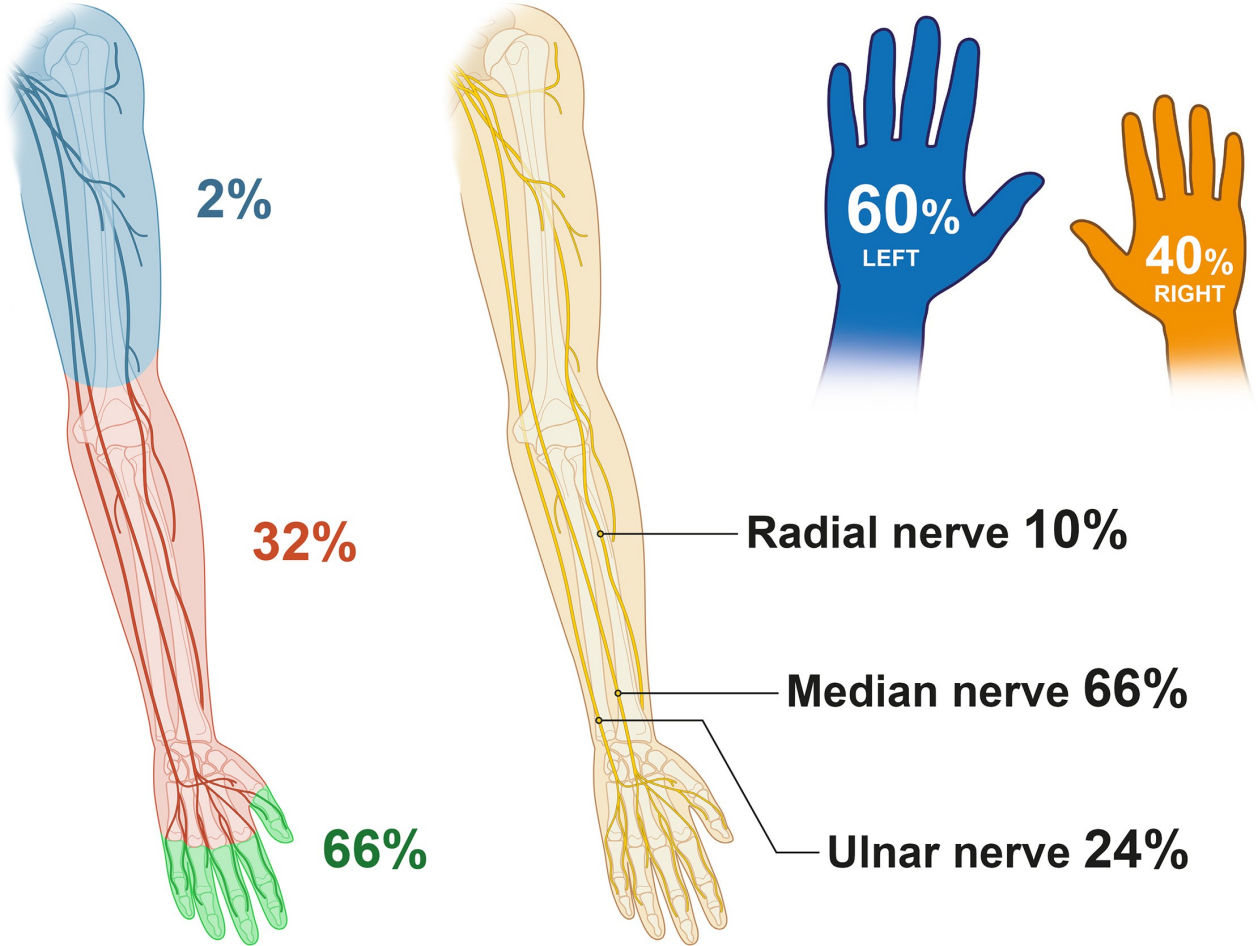
**Characteristics of nerve injuries: *Left*: Shown is the level of nerve injury as percentage of all included nerve injurie in a selected collective of work-related nerve injuries.** These demonstrate a mainly distal location of these nerve injuries. *Middle*: Shown is the proportion per nerve of all included single nerve injuries, with a majority of median nerve lesions. See Table 1 for detailed information on injured nerve in relation to injury location. *Right*: The majority of injuries was to the left hand.

Most patients (93%, N = 232) acquired single nerve injuries, of which 66% were median- (N = 153), 24% ulnar- (N = 56) and 10% radial nerve (N = 23; Fig 3, Table 1). Combined nerve injuries occurred most frequently to the median and ulnar nerve (94%, N = 17 of 18 total). Considering the entire population of injured nerves, the median nerve was affected in 63% (N = 170), the radial nerve in 27% (N = 73) and the ulnar nerve in 9% (N = 25). All but one injury was single hand only, with 60% being to the left hand (N = 151) and 40% to the right hand (N = 98; Fig 3). Also, in cases with multiple injured nerves, the right hand was more frequently injured (55% N = 10/18).

**Table 1:** Location of injury and relative probabilities to the entire sample. In detail the probability of injury to the three main nerves is described from proximal to distal. Likewise, the probability of combined nerve injury as well as concomitant vascular or tendon injury is described.

Probability of Injury			
Injury	Proximal to Wrist	Wrist and Metacarpus	Finger
**Median nerve**	26%	**50%**	**80%**
**Ulnar nerve**	**69%**	44%	24%
**Radial nerve**	15%	20%	0.01%
**Combined nerve injuries**	**11%**	10%	0.05%
**Tendon or vascular injury**	47%	60%	**79%**

Of all included nerve injuries, 11% (N = 29) were partial, thereof being 65% (N = 19) median nerve injuries and 24% (N = 7) ulnar and 10% (N = 3) radial. The rate of partial injuries per nerve was almost the same in all three nerves (median 12%, ulnar 10%, radial 12%), which was not statistically different (*P = 0*.*816*, Chi-Square Test). In partial nerve injuries, the mean extent of nerve damage was estimated macroscopically on average at 46%±34% (Mean±SD) of the nerve’s cross-sectional area (Fig 2).

Frequency of injury increased from proximal to distal, with 8% (N = 19) proximal to the wrist, 26% (N = 65) at the wrist and metacarpus, and 66% (N = 166) at phalangeal level (all relative to 250 injured patients, Table 1).

72% (N = 182) had concomitant vascular or tendon injury, with 49% (N = 123) having on average 2.3±1.9 tendons and 56% (N = 140) having on average 1.4 ±1 vessels injured. Most patients with combined injuries had the median nerve injured, which had tendon injury in 50% (N = 76) and vessel injury in 59% (N = 91; Table 2). Probability of concomitant injury was highest in lower arm nerve injuries (80%), followed by finger injuries (75%).

**Table 2:** Various aspects following acute treatment in relation to injured nerve, injury location and concomitant nerve, vascular or tendon injury. Interestingly, there is a significant increase for rehabilitation in patients with ulnar nerve injury and proximal nerve injuries. The surprisingly low number of rehabilitations in concomitant tendon or vascular injury is likely due to the high prevalence of very distal finger nerve injuries. Likewise, for nerve injuries proximal to the wrist, there is a high percentage of disability pension, which was however not quite statistically different. The column disability pension refers to percentage of reduced work ability, patients suffered as a consequence of the nerve injury. All P-Values were calculated using Kruskal-Wallis Test.

Post-Acute Treatment Variables								
Injury	Rehab	Sig.	Rehab—Duration days		Sick leave days		% receiving Disability pension	
Median nerve*	11%	***P = 0*.*002***	41±22	*P = 0*.*215*	133±136	*P = 0*.*599*	30%	*P = 0*.*581*
Ulnar nerve*	**23%**		30±9		160±213		25%	
Radial nerve*	13%		39±10		190±173		38%	
**Proximal to Wrist**	**36%**	***P = 0*.*007***	41±22	*P = 0*.*257*	203±294	*P = 0*.*524*	70%	P = 0.095
**Wrist & Metacarpus**	24%		47±20		125±123		36%	
**Finger**	16%		37±19		136±137		25%	
**Combined nerve injuries**	44%	*P* = 0.146	63	*P* = 0.388	102±117	*P* = 0.143	33%	P = 0.703
**Single nerve injuries**	21%		36±15		167±232		27%	
**Tendon or vascular injury**	18%	*P* = 0.862	40±21	*P* = 0.916	123±112	*P* = 0.734	30%	P = 0.353
**Isolated nerve injuries**	21%		36±15		167±232		20%	

* That this refers to single nerve injuries only.

### Acute treatment

83% (N = 207) of injuries were treated within 24 hours, the rest being secondary referrals. Treatment was either direct epineural repair 80% (N = 200), reconstruction with a nerve conduit 14% (N = 36) or using autologous nerve transplants for reconstruction 5% (N = 14). Mean duration of stay for acute treatment in hospital was 7±6 days, which was significantly higher (T-Test, p = 0.02) at 13±6 days if more than one nerve was involved.

### Cost analysis

Single median nerve injury averaged 7±5 days at the hospital and costs of 3.570±2.997€. Ulnar nerve injury averaged 5±3 days and costs of 2.650±1243€. Radial nerve injury averaged 6±4 days and costs of 3.166±1858€. Compared to single nerve injury, averaging 3.308±2.628€, combined nerve injuries were significantly (p>0.01; T-Test) more expensive at 7.962±6.896€. Cost increased as well, if nerve injuries were combined with tendon 5.086€ or vascular injury 4.886€.

Nerve injuries proximal to the wrist averaged 9±9 days at the hospital on average and cost on average 5.360±6.429€. At the wrist and metacarpus, they averaged 7±4 days at the hospital costing 3.534±2.710€. At the phalangeal level, average hospital stay was 7±6 days and costs 3.418±3.330€.

### Inpatient rehabilitation

A total of 16% (N = 40) of patients underwent inpatient rehabilitation, of which 60% had a single, 25% two and 15% three separate rehabilitation stays. The average time of inpatient treatment was in sum 41±21 days with combined costs of 5.842±2.451€ per patient, which increased from one (4.141±1813€) to two (6.981±3.372€) to three (10.750±3.501€) stays. Compared to patients without inpatient rehabilitation averaging 3.358±3.274€, the overall hospital costs were significantly (p>0.001; T-Test) higher at 12.294±6.366€.

### Sick leave

Average sick leave was 147±163 (Min: 11; Max: 1109) days with socioeconomic costs of 197€/day [14]. Therefore, the total average per person was 17.640€, further increasing the average costs per patient to 32.526± 24.117€ (Table 2). 26% required a gradual reintegration program into work, lasting on average 21±22.96 (Min: 7 Max: 122) days. In 4%, the patient had to be reintegrated into a different work situation and in 2% a retraining into a different profession was required as a consequence of the injury. Sick leave was on average 160±212 (Min: 14 Max: 1109) days for patients undergoing in-patient rehabilitation and 136±134 (Min: 11 Max: 587) days for patients undergoing out-patient rehabilitation and did not statistically differ from each other (*P = 0*.*289; Mann-Whitney U Test*).

### Disability & pension

Overall, 38% of the patients with long-term follow-up were assessed in a medical estimate by a trained surgeon. Here it was determined if they suffered from a substantially reduced ability to work following the nerve injury. In 30% of the total number of patients, the impact of the injury was deemed to reduce their ability to work for at least 20%. This is the minimum for a financial compensation in Germany and subsequently these patients received continuous payments. In the patient cohort the average reduction was 22%, thus requiring a continual compensation which was on average 3188±2651€ annually (Table 2). In 7% the severity of the impairment required support for the patient in daily routine situations at home or in social life (e.g. household aid), thus further increasing lifelong costs of the injury on average by 16.872± 12632€ per year.

## Discussion

Peripheral nerve injuries of the upper extremity produce significant socioeconomic costs, regardless of location or clinical appearance[15, 16]. Even small injuries at the phalangeal level, especially at the thumb and index finger, can lead to significant morbidity and costs for initial treatment and if disabilities persist, life-long compensation. This is evident from our long-term costs-analysis, that indicated that 30% of patients suffering from work related traumatic nerve injuries had permanent disabilities and received financial compensation. Considering the current average life expectancy in Germany of this patient generation (1976) of 72 years [17] and their average age at injury being 39.9 years, this results in 32.1 years that have to be compensated with approximately 102.167€ per patient. Based on our calculations, these 30% of patients suffering from severe injury would total an overall socioeconomic burden of 138.798€ € for acute treatment, rehabilitation and life-long compensation or even 155.670€ when requiring gradual reintegration. This number will continually rise as life expectancy for Germans born in 2019 is calculated at 80.8 years, thus increasing overall compensation to 130.061€ and overall costs to 166.692€, if these patients were born in 2019[17].

While other traumatic injuries as for example polytrauma are generally more expensive[18, 19], it is the frequency of hand and upper extremity injuries and the subsequent risk of nerve injury that constitutes its socioeconomic importance, especially in work related accidents. In a recent analysis from the German Trauma Registry (TraumaRegister DGU®), 3.3% of all trauma patients (private and work accidents) with upper limb affection suffered from additional peripheral nerve injury, and in a subset of motor cycle injuries this increased to 32.5%[1]. This study likewise showed that trauma patients with nerve injury compared to equally injured patients without, had significantly longer primary hospital stay of on average six days and required more inpatient rehabilitation[1]. Both duration of stay and long-term rehabilitation were indicators for higher costs and associated with longer time off work. Studies from other countries found a similar epidemiology for populations of private and work accidents[20, 21], thus indicating that the results from our study may well be translatable to private accidents. Compared to other frequent hand injuries, as for example the distal radius fracture, the costs of nerve injuries are either comparable or often higher. For example, palmar plate osteosynthesis in distal radius fractures are approximately 10 to 30% less expensive than injuries to either of the three main nerves of the hand (even distal digital nerve injuries) in the German health system [22].

As a consequence of the socioeconomic costs, the German Social Accident Insurance has highlighted the importance of peripheral nerve injuries of the upper extremity by labeling all injuries to the main nerves and the digital nerves responsible for functionally-relevant hand sensibility (digital nerves 2, 3 and 10) as most severe type of injury[23]. These phalanges are at the same time the most-frequently injured ones[11]. In Germany, it is mandatory that all these injuries are treated at specialized trauma centers that are certified to provide the highest standard of care and specialization in peripheral nerve surgery in order to minimize the risk of long-term morbidity and secondary complications. This is an effort to reduce the high socioeconomic costs of nerve injury, as are for example caused by sick leave which was on average almost five months in this cohort. Furthermore, 26% of these patients required gradual reintegration for on average 21 days, until fit for full employment. Yet, it is important to highlight that 9% of our patient cohort had sick leaves extending beyond a year, and other studies showed ratios of up to 41%[2]. These long sick leaves may result from the majority of patients (60%) being injured in trades that are associated with manual labor, such as construction or the wood and metal industry. Thereby results the susceptibility to nerve injuries, especially at the hand or phalangeal level, while heavy objects or heavy machinery are operated on. Even a small injury with affection of a digital nerve and consecutively lost sensibility, or worse a lost ability to trust the sensibility or force production of the hand in more proximal injuries, is often a substantial obstacle to return to these professions. Thus, in 4% of cases, patients had to be reintegrated into a different work situation and 2% even underwent retraining to a profession feasible despite the sustained permanent disability. These numbers are comparable to a previous study from Sweden, where 8% of patients (private and work accidents) with digital nerve injuries were not able to return to their previous work[11]. Especially, while accompanying these patients through their treatment, surgeons witness the massive consequences of a hand not trusted or felt properly, and its secondary consequences on psychological well-being, private life and the fear of losing employment. Similar is the experience in permanently disabled patients that we see for determining their loss of function after the end of treatment, where the dire sequelae of even small nerve injuries are often most evident. Psychological consequences of nerve injuries have been well-studied and affirm these observations[4, 24, 25], and may sometimes incorrectly be interpreted as an exaggeration of a seemingly small injury.

The patient selection on work accidents in this study results from the very detailed analyses of these patients by the German Social Accident Insurance as well as providing these follow up data, which is not the case for private injuries. It may be considered as a limitation and difficulty to translating its conclusions to all patients. However, while focusing on work-related accidents may select certain aspects, the overall patient characteristics were very similar to other studies without this selection. Furthermore, these patients are well observed throughout their entire treatment and thus provide a unique and unified data set, that ultimately enabled this complex analysis. In addition, the selection of primary traumatic nerve injuries and the exclusion of secondary nerve injuries (fracture-related or of iatrogenic origin) provides a well-defined cohort, that would otherwise suffer from a very heterogenic patient population, such as for example long sick leaves due to fracture healing.

A very interesting aspect of this study is that predominantly peripheral nerves of the left hand were injured (60%). Studies indicate that more 80–90% of the population are right-handed[11, 26], thus leading to the assumption that the left injured hand was used to hold an object for manipulation in these cases. As a large number of nerve injuries were caused by electrical machines such as electrical drills or saws, where the non-dominant hand is used for securing the object, this further emphasizes this conclusion and highlights a potential for injury prevention. As our analyses indicate, there are specific tools and environments that have an increased risk for upper extremity nerve injury, which may be improved and thus the injury mechanisms preventable. European Hand surgery societies (FESSH) have started programs to raise awareness for the underlying causes of hand injuries and thereby prevent the before mentioned injury mechanism[26]. As these measures have only recently been introduced, their impact is yet unknown. Given the costs of nerve injury and the subsequent effects on patient’s ability to work, it is however very likely that funding towards prevention, specialized treatment and rehabilitation is well spent.

## Conclusion

This study is the first to provide a detailed analysis of the associated costs of upper extremity nerve injuries in a large German population. Our data indicate high costs, long-term sick leaves and substantial permanent disability in the studied population for both, proximal and distal nerve injuries. This data is important to correctly assess the magnitude of such injuries and specifically provide knowledge on the economic and social consequences. Based on this information, new strategies on prevention and treatment can be developed and thus help to define specific guidelines for peripheral nerve injuries of the upper extremity.

## Supporting information

S1 Checklist CHEERS checklist. (PDF)

S1 Data (XLSX)

## List of abbreviations

DRG: Diagnosis related groups system
